# Karyological, morphological and phytochemical characteristics of medicinal plants *Sophora flavescens* Aiton grown from seeds collected at different localities

**DOI:** 10.1186/1999-3110-55-5

**Published:** 2014-01-16

**Authors:** Tzu Che Lin, Jih Min Sung, Mau Shing Yeh

**Affiliations:** 1 Department of Agronomy, National Chung Hsing University, Taichung, ROC Taiwan; 2 Department of Food Science and Technology, Hungkuang University, Shalu, Taichung, ROC, Taiwan

**Keywords:** Antioxidant activity, Karyotype, Matrine, Oxymatrine, *Sophora flavescens* Aiton, Total phenol

## Abstract

**Background:**

Dried roots of *Sophora flavescens* Aiton contain many phytochemicals that exhibit beneficial effects on human health. This study examined and compared the karyological, morphological and phytochemical characteristics of three *S. flavescens* populations collected from the Danda, Hualien and Yuli of Taiwan and a population collected from Gansu, China.

**Results:**

Karyotypes of the four populations were similar, with a diploid number of 2n = 18. The Hualien population produced more roots but with less matrine and oxymatrine contents in its root tissue than others. However, only the root of Danda population had a measurable level of naringenin. The dried root of Yuli population had greater ferric reducing antioxidant power and trolox equivalent antioxidant capacity than that of the other populations.

**Conclusions:**

Thus, the collected *S. flavescens* populations, particularly the population collected from Danda, have the potential to be used in breeding programs.

**Electronic supplementary material:**

The online version of this article (doi:10.1186/1999-3110-55-5) contains supplementary material, which is available to authorized users.

## Background

*Sophora flavescens* Aiton is a traditional Chinese medicinal herb of the Fabaceae family with wild populations distributed mainly in East Asia. The dried roots of *S. flavescens,* which contain many alkaloids such as matrine and oxymatrine (Liu et al., 2011), are commonly used to treat viral hepatitis, cancer, viral myocarditis, gastrointestinal hemorrhage and skin diseases (Chen et al., 2004; Ling et al., 2007; Cao et al., 2011). They also contain many flavonoids that have antioxidative potential and beneficial effects on human health (Piao et al., 2006; Jeong et al., 2008). *S. flavescens* is widely used in Taiwan, but the wild populations of *S. flavescens* grown in native vegetations are very limited. Therefore most *S. flavescens* plant materials are imported from China to meet the market demand. However, wild *S. flavescens* populations in China are threatened by over-harvesting (Zhao et al., 2004), thus the commercial-scale cultivation of *S. flavescens* is highly desirable.

In this study, seeds of *S. flavescens* collected from three different localities in Taiwan and a locality in China were planted at the experimental farm of National Chung Hsing University (NCHU) (Taichung, Taiwan). Several morphological characteristics (plant height, number of primary branches, seed weight and root dry weight) and the microscopic observations of root, stem and leaf tissues, as well as some phytochemical characteristics (total phenol, flavonoids, matrine, oxymatrine and antioxidant activity) of field-grown *S. flavescens* populations collected from different localities were compared. The karyological information is useful for in species identification and analysis of hybrid populations (Michetti et al., 2010; De Souza Almeida et al., 2007), and currently little information on karyotypes of the *S. flavescens* species is available even though it is reported to be a diploid (2n = 18) (Marhold, 2009). Therefore, attempts were also made to examine detailed karyotypes of the four populations. The observed variations should provide useful information concerning the potential value of these *S. flavescens* populations in producing functional substances.

## Methods

### Plant materials

The seeds of *S. flavescens* collected from the native vegetations of Danda, Hualien and Yuli regions of Taiwan were planted at the experimental farm of Department of Agronomy, NCHU (Taichung, Taiwan). Seeds of *S. flavescens* were also collected from Gansu, China and plants grown at NCHU were used for comparisons.

### Karyological analysis

For karyological examinations, the seeds of 4 *S. flavescens* populations were soaked in tap water for 24 h. They were then put in Petri dishes lined with filter paper and were regularly watered. Petri dishes containing seeds were kept in the dark at 25°C. Root-tips were collected when the primary roots were 1–3 cm long and were excised and pretreated at room temperature for 4 h in 0.2 mM 8-oxychinolin in the dark. After pretreatment the root tips were fixed in a solution of 95% alcohol: glacial acetic acid (3:1, v/v) solution for 24 h. The chromosomes were stained following the procedures as described by Sharma and Gupta (1982) after hydrolysis in 1 N HCl for 10 min at 60°C. The root-tips (0.5 cm) were transferred to a fresh Feulgen solution for 30 min and then to distilled water for another 5 min (Kasten, 1956). The meristematic part was used for microscope examination. Chromosomes were examined at 1000 x magnification using a microscope (BX 50, Olympus, Tokyo, Japan) equipped with a photographic attachment (E330, Olympus, Tokyo, Japan). The paired homologous chromosomes were arrayed in descending order of length. Chromosome measurements including long arm, short arm, chromosome lengths, arm ratio index, percentage of relative length and chromosome type (Levan et al., 1964) were taken from enlarged well-spread metaphase cells obtained from 10 root-tip samples of each population.

### Morphological examinations

The collected *S. flavescens* populations were grown at the experimental farm of NCHU using the common practices. The experiment was set up following a randomized complete block design with four replicates. The seeds were sown in the nursery plots in August, 2008. The grown seedlings were then transplanted to the experimental plots. Three seedlings per hill were planted with a spacing of 85 cm x 50 cm in each experimental plot of 10 m x 3 m. Ten of one-year-old plants from each experimental plot were tagged and used for agronomic characteristics (e.g., plant height, number of roots per plant and root dry weight) observations. For microscopic observations, the samples of *S. flavescens* roots (5–10 cm from tap root base), stems (the fourth or fifth node from shoot apex) and leaves (the leaflet subtended on the fourth or fifth node from the shoot apex) were fixed in a FAA (formalin/glacial acetic/70% ethanol in the ratio of 0.5:0.5:9.0) and dehydrated through the gradual ethanol series and *t*-butanol. These samples were then buried in paraffin, and sectioned with a microtome (Leica 1515, Leica Instruments, Nussloch, Germany) into slices of 15 μm thickness. The section tissues were stained with a safranin-fast green solution, and then used for microscope examination (BX 50, Olympus, Tokyo, Japan).

### Phytochemicals determinations

Ground root materials (1 g) sampled from each of the replicates prepared from four *S. flavescens* population were extracted with 20 ml methanol (80:20, v/v in H_2_O) for 15 min, and the filtrate volume was re readjusted to 20 ml with extraction reagent. The contents of total phenols in extracts were estimated using the Folin-Ciocalteau assay with a Hitachi U-2900 spectrophotometer (Tokyo, Japan) (Aguilar-Garcia et al., 2007). The absorption value was determined at 735 nm, and gallic acid was used for constructing the standard curve. The flavonoids contents in extracts were measured using the aluminum chloride colorimetric assay as described by AbouZid and Elsherbeiny (2008). The contents of matrine and oxymatrine (alkaloids) were analyzed using high performance liquid chromatographic method as described by Li and Wang (2004). The chromatography system consisted of a high performance liquid chromatography (HPLC) (Hitachi 7200, Tokyo, Japan) equipped with an Inertsil ODS-2 column. The mobile phase comprised of A: methanol-10 mM KH_2_PO_4_-triethylamine in a ratio of 96:6:0.01 (v/v/v) and B: methanol. The injection volume was 20 μl, the flow rate was 1.0 ml min^-1^, and the UV wavelength was set at 210 nm. Standards of matrine and oxymatrine were obtained from Wako Company (Tokyo, Japan).

### Antioxidant activities determinations

To measure the ferric reducing antioxidant power (FRAP), a portion of the root extract (0.5 ml) used for phytochemical determinations was mixed with 0.5 ml of 0.2 mM sodium phosphate buffer (pH 6.6) containing 1.0% K_3_Fe(CN)_6,_ and the mixture was incubated at 50°C for 20 min. After adding 0.5 ml of 1% trichloroacetic acid (w/v), the mixture was centrifuged at 200 g for 10 min. The upper layer (0.25 ml) was mixed with 3.75 ml of de-ionized water containing 1% ferric chloride, and the absorbance was monitored at 700 nm in a spectrophotometer (Jeng et al., 2010).

The trolox equivalent antioxidant capacity (TEAC) assay is based on the scavenging of 2,2'-azinobis-(3-ethylbenzothiazoline-6-sulfonic acid)-radical (ABTS^-.^). A solution of ABTS^•^ was prepared in 0.1 M saline phosphate buffer (pH 7.4, 0.15 M sodium chloride) by mixing 2.5 mM of AAPH with 2.0 mM of ABTS^2−^ The solution was then heated for 16 min at 60°C, protected from light, and stored at ambient temperature until used. To measure the radical scavenging activity, 40 μl of the root extract was mixed with 1.96 ml of ABTS^-.^ solution. Absorbance of the solution was monitored at 734 nm, and trolox was used in the construction of the standard curve (Jeng et al., 2010).

### Statistical analysis

The karyological examinations were conducted on the 10 root tips samples randomly selected from each of the *S. flavescens* populations. The morphological and phytochemical and antioxidant activities determinations were conducted on the field-grown *S. flavescens* populations. Data were analyzed by using the Statistical Package for Social Science (SPSS 10.0 for Windows: SPSS INC., Chicago, Il, USA). Values were given as mean ± standard deviation and means were compared using a least significant difference (LSD) test.

## Results and discussion

### Karyological characteristics

**Figure 1:**
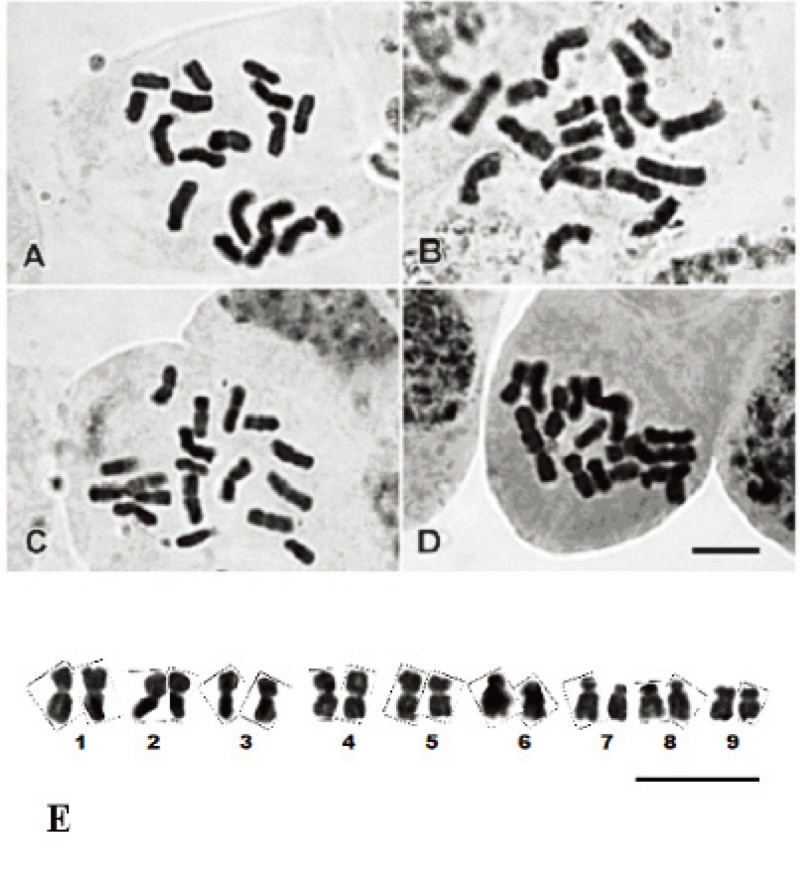
**Mitotic metaphase chromosomes of** ***S. flavescens*** **populations collected from (A) Gansu, China, (B) Danda, Taiwan, (C) Hualien, Taiwan, (D) Yuli, Taiwan, scale bar = 10 μm, and (E) karyotype of** ***S. flavescens*** **population collected from Yuli, Taiwan.** Scale bar = 10 μm.

The apical meristems of plant roots are frequently used to examine mitosis division and identification of chromosome arrangement, because root induction occurs rapidly and mitosis division is fast in this area (Safarnejad and Hosaini, 2005). Therefore, in the present study, root tips were used for comparing karyotypes of four *S. flavescens* populations. As presented in Figure 1, mitotic chromosome counts showed a diploid number of 2n = 18 for all the *S. flavescens* populations collected from different localities. These results coincide with the previous report of Marhold (2009). Moreover, normal karyotype without satellites was observed in karyotype of populations.

Karyological characteristics of all the examined populations were similar. These results are not surprising, because the molecular assessments on internal transcribed spacer sequence showed that the tested *S. flavescens* populations have extremely low genetic variability (Lin et al., 2012). Since the karyological characteristics for the tested populations were similar, therefore, the karyotype data were presented as the means of the tested four populations (Table 1). In general, the karyotype of *S. flavescens* consists of two pairs of submetacentric chromosomes (chromosome pairs 7 and 8), with the arm ratio index greater than 1.71 (Table 1), according to the classification of Levan et al. (1964). The others five chromosome pairs were metacentric, with the arm ratio index ranged from 1.30 to 1.63 (Table 1). The total length of chromatin was 40.38 μm, the longest and shortest chromosomes were estimated 5.66 μm (chromosome pair 1) and 3.68 μm (chromosome pair 9), respectively (Table 1).

**Table 1:** **Measurements of karyological characteristics of** ***S. flavescens*** **populations** ^**a**^

Chromosome pair	Short arm (μm)	Long arm (μm)	Total length of chromosome (μm)	Arm ratio index	Percentage relative length (%)	Chromosome type
**1**	2.15 ± 0.29	3.51 ± 0.31	5.66 ± 0.70	1.63	7.01	m
**2**	2.05 ± 0.15	2.98 ± 0.41	5.03 ± 0.46	1.45	6.23	m
**3**	2.12 ± 0.46	2.75 ± 0.39	4.87 ± 0.55	1.30	6.03	m
**4**	1.96 ± 0.24	2.73 ± 0.38	4.69 ± 0.47	1.39	5.81	m
**5**	1.85 ± 0.21	2.75 ± 0.37	4.60 ± 0.56	1.49	5.70	m
**6**	1.69 ± 0.24	2.48 ± 0.24	4.17 ± 0.44	1.46	5.16	m
**7**	1.47 ± 0.25	2.51 ± 0.15	3.98 ± 0.36	1.71	4.93	sm
**8**	1.48 ± 0.14	2.23 ± 0.21	3.70 ± 0.24	1.87	4.58	m
**9**	1.28 ± 0.26	2.40 ± 0.31	3.68 ± 0.35	1.51	4.56	sm
**Total**	16.04	24.34	40.38			

*Abbreviation*: *m* metacentric, *sm* submetacentric.

^a^Results are shown as mean ± standard deviation (n=10).

### Morphological characteristics

**Figure 2:**
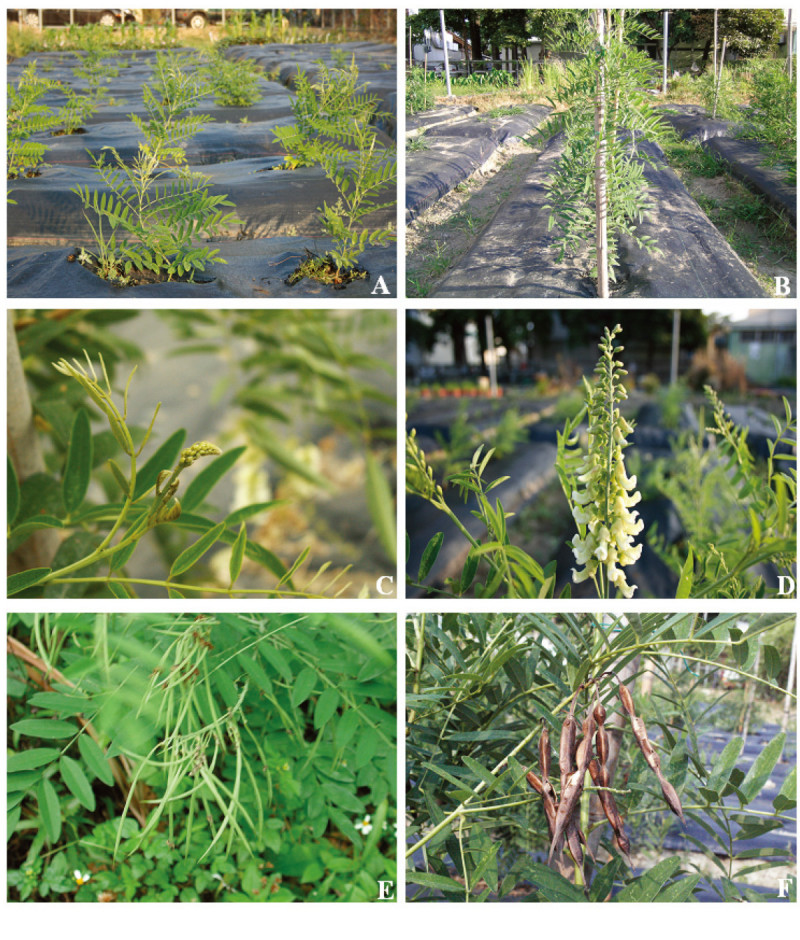
**Field-grown** ***S. flavescens*** **plants (Yuli population). (A)** early phase of plant growth, **(B)** mid phase of plant growth, **(C)** flower bud formation, **(D)** flower petals expantion, **(E)** pod formation and **(F)** mature pods.

All the *S. flavescens* populations grew vigorously at the experimental farm of NCHU located in Taichung city, even though they were grown from seeds collected from different localities (Figure 2). However, significant differences in the agronomic characteristics were found among the populations (Table 2). The highest number of primary branches produced per plant was obtained from Yuli population, followed by the Hualien, Gansu (China) and Danda populations. Plants of the Danda population produced more flowers and pods per inflorescence, and more seeds per pod, but have less 100-seeds weight, than other three populations (Table 2). On the other hand, the plant of Hualien population produced more roots than others.

**Table 2:** **The morphological characteristics of** ***S. flavescens*** **populations** ^**a**^

Characteristics	Gansu	Hualien	Yuli	Danda
**No. of leaflet**	17.00 ± 1.90 ab	19.00 ± 3.47 a	17.00 ± 2.36 ab	15.00 ± 2.83 b
**Leaflet length (mm)**	33.63 ± 4.62 b	42.40 ± 4.97 a	43.12 ± 4.92 a	41.61 ± 4.89 a
**Leaflet width (mm)**	10.88 ± 1.82 bc	12.13 ± 1.61 ab	13.37 ± 1.32 a	9.56 ± 0.93 c
**No. of primary branches**	3.78 ± 0.42 b	4.67 ± 0.93 ab	5.00 ± 0.76 a	1.89 ± 0.19 c
**No. of flower per inflorescence**	33.38 ± 0.88 b	34.34 ± 1.03 b	31.00 ± 1.45 c	43.20 ± 3.23 a
**No. of pod per inflorescence**	2.50 ± 0.58 c	5.67 ± 1.57 b	6.05 ± 0.97 b	9.05 ± 2.51 a
**No. of seed per pod**	7.84 ± 0.69 ab	7.04 ± 0.71 b	7.95 ± 0.84 ab	8.59 ± 0.27 a
**100-seed weight (g)**	4.02 ± 1.07 b	4.11 ± 0.44 b	4.60 ± 0.52 a	3.60 ± 0.55 c
**Dried root weight (g)**	142.45 ± 26.94 bc	284.66 ± 41.40 a	191.26 ± 30.02 b	122.40 ± 25.54 c
**No. of roots per plant**	6.40 ± 2.07 a	5.20 ± 1.64 a	7.60 ± 2.06 a	7.25 ± 1.58 a
**Plant height (cm)**	73.06 ± 19.37 b	107.32 ± 18.03 a	86.00 ± 18.16 ab	75.75 ± 25.30 b

^a^Means ± standard errors (n = 4) within each column followed by different letters are significantly different at the P = 0.05 level.

### Microscopic observations

**Figure 3:**
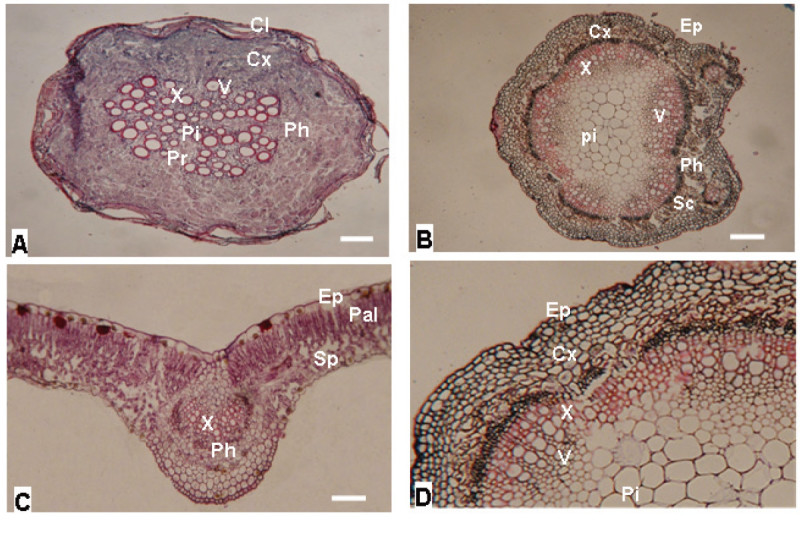
**Cross sections of (A) root, (B) stem, (C) leaf and (D) enlarged stem cross section of** ***S. flavescens*** **plants (Yuli population).** Cx: cortex, Cl: cork layer, Pi: pith, Pr: pith ray, Ph: phloem, V: vessel, X: xylem, Ep: epidermis, Pal: palisade tissue, Sc: sclerenchyma, Sp: spongy tissue, Bar = 5 μm.

The transverse sections of roots, stems and leaf tissues of *S. flavescens* populations are similar within each tissue, therefore, only the tissue samples of Yuli population were illustrated in Figure 3A-D. The mature root is almost circular in outline (Figure 3A). Cork is multilayered with a broad and well developed zone comprising of 9 (Yuli) to 12 (Danda) layers (data were determined from enlarged root cross section that were not shown in Figure 3), and the cells are flat in shape. However, cork often split and dropped. Cortex consists of 22 (Yuli) to 28 (Danda) layers (data not presented) of parenchyma cells. Cambium is a distinct zone of two (Yuli) to six (Danda) layers of small meristematic cells. There are 12 (Yuli) to 17 (Gansu) pith rays (data not presented).

The epidermis of *S. flavescens* stem is single layered with outer cuticle (Figure 3B). The cortex consists of 6 (Gansu) to 11 (Hwalien) layers (Figure 3D) of round cortical parenchyma cells. The pith is large and consists of large parenchyma cells.

There is a single layered epidermis on the upper and lower surfaces of the leaf (Figure 3C). The palisade parenchyma in contact with the upper epidermis consists of one to two layers of relatively elongated cells, while the spongy parenchyma in contact with lower epidermis has two to three layers. In the median region of the leaf, there is a large vascular bundle.

### Phytochemicals

Phenols constitute one of the most abundant groups of secondary metabolites, ranging from simple molecules (e.g., phenolic acids) to highly polymerized compounds (e.g., lignins), in plant kingdom. They have been demonstrated to prevent the development of many chronic diseases, which might be associated with their powerful antioxidant and free radical scavenging properties (Manach et al., 2004). In the present study, the amounts of total phenols in the dried roots of *S. flavescens* varied from 32.38 (Hualien population) to 53.22 mg gallic acid g^-1^ (Danda population) on dry weight (DW) base (Table 3). The values are considerably higher than the total phenols content (20.8 mg gallic acid DW g^-1^) reported by Cai et al. (2004). This could be due to the different *S. flavescens* populations used between the two studies.

**Table 3:** **Comparisons of total phenols, flavonoids, matrine, oxymatrine, ferric reducing antioxidant power (FRAP) and trolox equivalent antioxidant capacity (TEAC) in roots of** ***S. flavescens*** **populations** ^**a**^

Population	Total phenols	Flavonoids	Matrine	Oxymatrine	FRAP	TEAC
	(mg g^-1^DW)	(mg g^-1^DW)	(mg g^-1^DW)	(mg g^-1^DW)	(mg g^-1^DW)	(mg g^-1^DW)
Gansu, China	41.49 ± 2.97 b	4.37 ± 1.12 b	19.72 ± 3.13 a	4.26 ± 0.82 a	19.52 ± 1.89 bc	314.90 ± 8.93 b
Danda, Taiwan	53.22 ± 4.10 a	6.48 ± 0.58 a	13.99 ± 2.60 ab	1.72 ± 0.38 b	25.01 ± 1.72 a	283.57 ± 11.81 c
Yuli, Taiwan	35.31 ± 3.85 c	3.17 ± 1.83 c	12.75 ± 2.83 b	1.14 ± 0.44 bc	23.20 ± 3.19 ab	364.06 ± 10.62 a
Hualien, Taiwan	32.38 ± 4.67 c	2.39 ± 1.14 c	10.14 ± 1.74 b	0.61 ± 0.16 c	19.44 ± 3.59 c	365.42 ± 9.48 a

^a^Means ± standard errors (n = 4) within each column followed by different letters are significantly different at the P = 0.05 level.

Flavonoids are a subclass of phenols, many of which can alter metabolic processes and have a positive impact on health (Beecher, 2003). The flavonoids regularly introduced with the diet can act as mild pro-oxidants and stimulate the endogenous antioxidant defenses. As shown in Table 3, the contents of flavonoids in the dried roots of *S. flavescens* populations ranged from 2.39 (Hualien population) to 6.48 (Danda population) mg g^-1^ DW. These values are considerably higher than that (0.79 mg g^-1^ root DW) reported by Gou et al. (2011).

The dried roots of *S. flavescens* also contain about twenty kinds of alkaloids, among them matrine and oxymatrine are the main effective constituents used for disease treatments (Wang et al., 2012). Therefore, only matrine and oxytraine were measured in this study. The matrine and oxymatrine contents were found to be significantly different among the examined *S. flavescens* populations (Table 3). The population from Gansu (China) had the highest amounts of matrine and oxymatrine, and then followed by the populations from Danda, Yuli and Hualien. Despite the Hualien population produced the highest amount of dried root (Table 2), its dried roots had the lowest levels of matrine and oxymatrine among the examined populations (Table 3).

### Antioxidant activities

Several techniques, such as 2,2-diphenyl-1-picrylhydrazyl (DPPH) radicals scavenging, ferric reducing antioxidant power (FRAP) and lipid peroxidation inhibition ability, have been used to determine anti-oxidative activities in selected foods, but each with different specificity and sensitivity (Frankel & Meyer, 2000). In this study, both FRAP and Trolox equivalent antioxidant capacity (TEAC) were applied to evaluate the anti-oxidative capacity in the roots of *S. flavescens* population. Statistical analyses showed that the *S. flavescens* populations collected from Danda tended to have relatively greater FRAP activity than the other three populations (Table 3). On the other hand, the highest TEAC activity was obtained from the Hualien population, and then followed by Yuli, Gansu and Danda populations (Table 3). Both FRAP and TEAC are electron transfer-based assay, in which the antioxidant action is simulated by allowing the antioxidants to react with a suitable redox-potential probe (fluorescent or colored oxidizing agent). Nevertheless, FRAP assay does not measure thiol antioxidants such as glutathione (Badarinath et al., 2010). Thus, this difference might partially illustrate why different ranking orders in anti-oxidative capacity were observed among the tested *S. flavescens* populations by using FRAP and TEAC.

## Conclusions

The *S. flavescens* populations used in this study were grown from seeds harvested from four different localities. No within-species karyotypic variations were observed among them. All the examined populations have a diploid number of 2n = 18, which indicated that the collected populations are cytologically stable. Significant morphological differences were found among the populations; with the population from Hualien produced more roots than the others. However, the lowest levels of matrine and oxymatrine were obtained from the dry root of the Hualien population. The dry root of the population from Gansu (China) had the highest amounts of matrine and oxymatrine. On the other hand, the roots of the Yuli population had the highest ferric reducing antioxidant power and trolox equivalent antioxidant capacity among the examined populations. Thus, the collected *S. flavescens* populations that show diversity in pharmaceutical and medicinal characteristics have the potential for breeding high-value *S. flavescens* populations.

## Authors’ original submitted files for images

Below are the links to the authors’ original submitted files for images. Authors’ original file for figure 1 Authors’ original file for figure 2 Authors’ original file for figure 3
