# Supplementary figures and images for: Karyological, morphological and phytochemical characteristics of medicinal plants Sophora flavescens Aiton grown from seeds collected at different localities
Source: Bot Stud. 2014 Jan 16;55:5. doi: 10.1186/1999-3110-55-5 (PMC5432817; doi:10.1186/1999-3110-55-5)

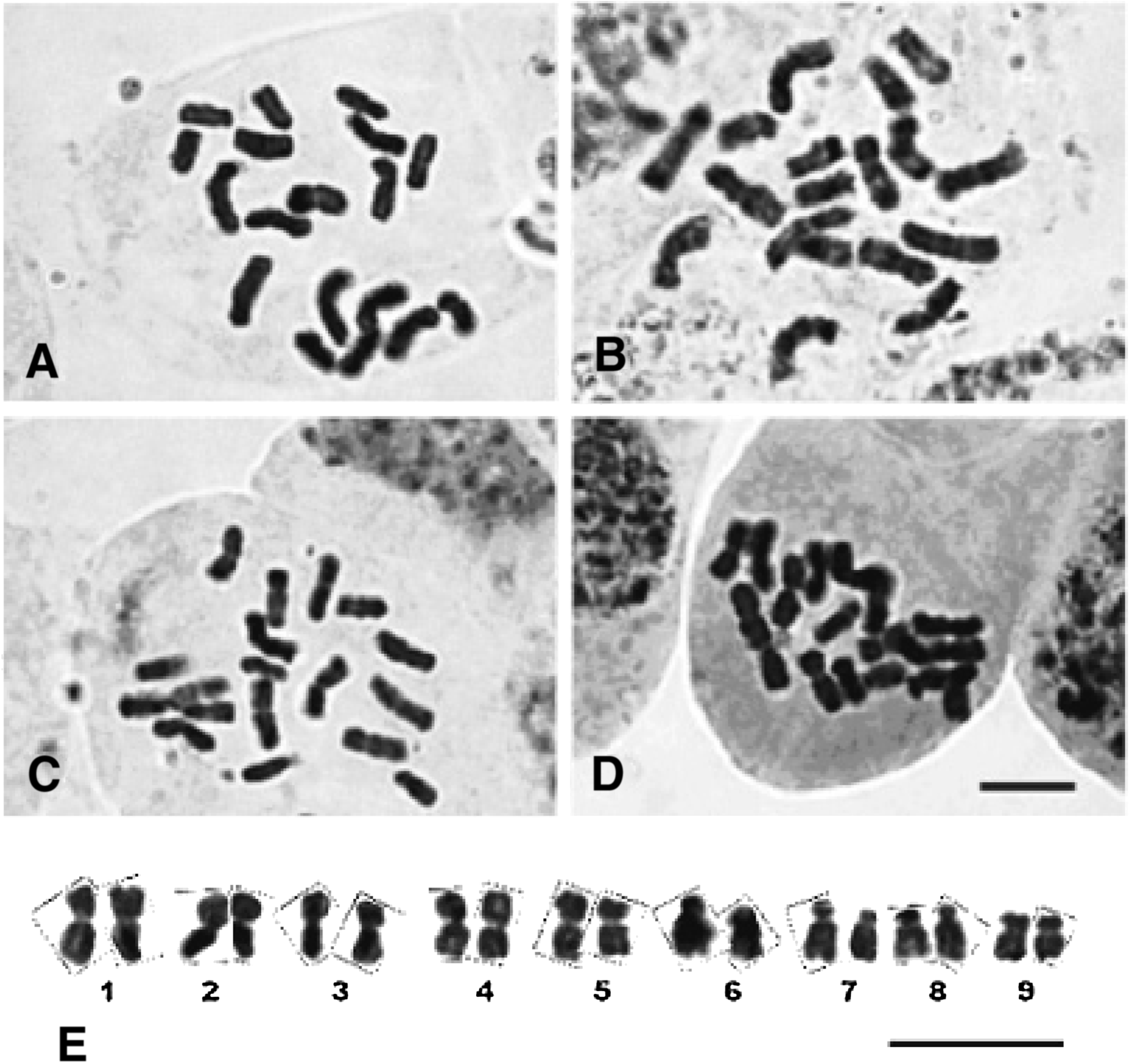

Supplement: Supplementary file 1 — Authors’ original file for figure 1 [file 40529_2012_58_MOESM1_ESM.tif]

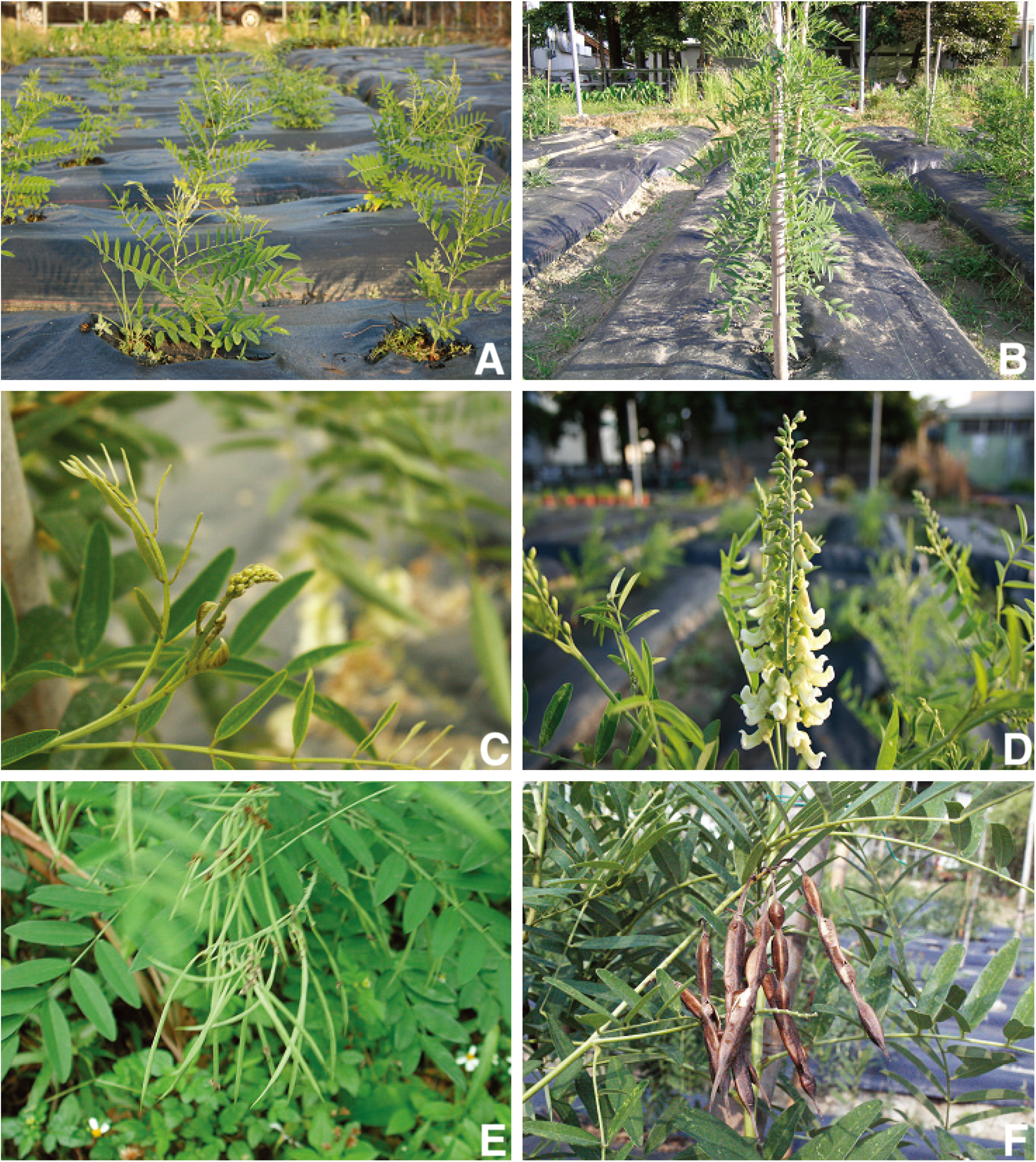

Supplement: Supplementary file 2 — Authors’ original file for figure 2 [file 40529_2012_58_MOESM2_ESM.tif]

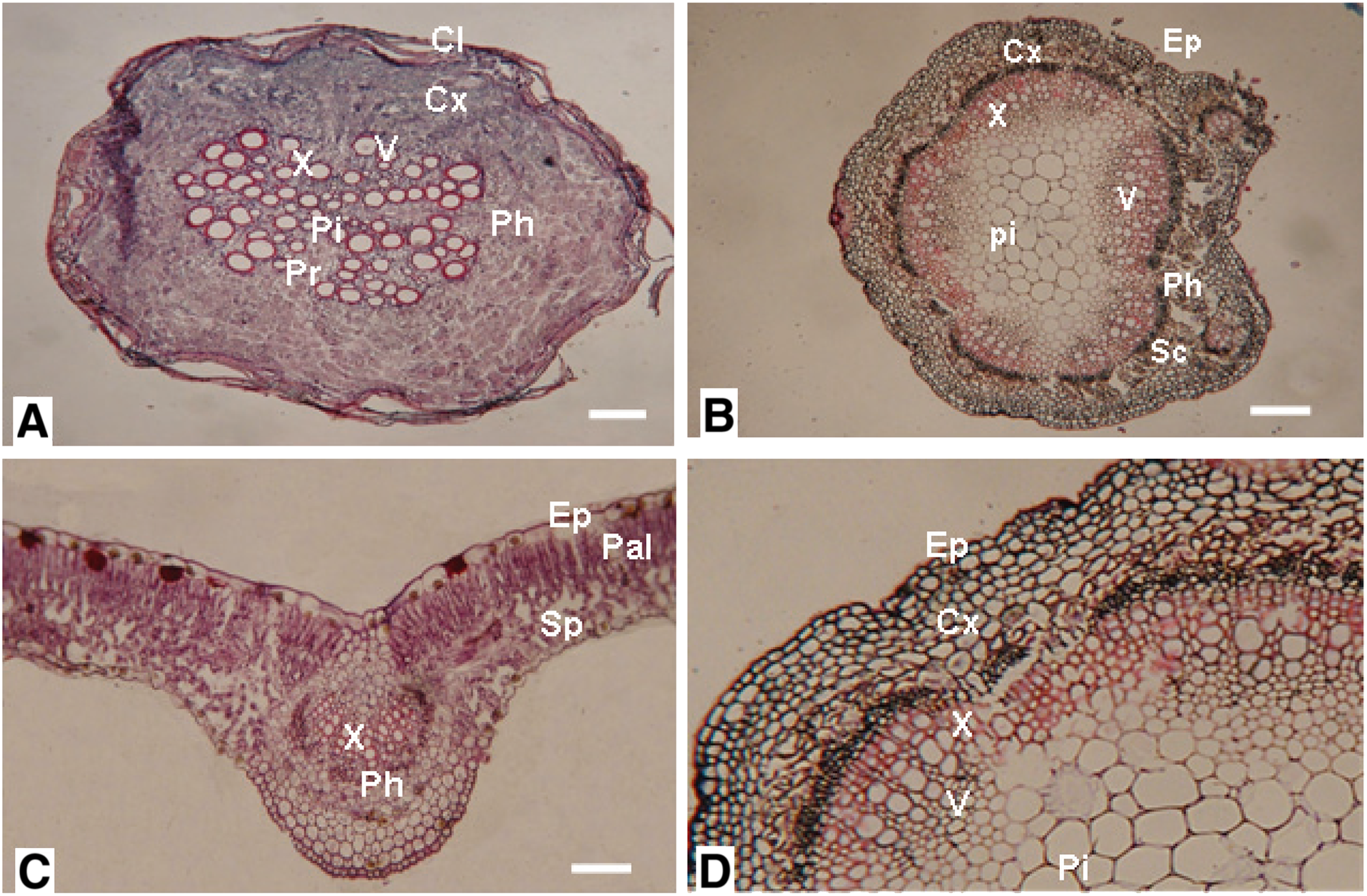

Supplement: Supplementary file 3 — Authors’ original file for figure 3 [file 40529_2012_58_MOESM3_ESM.tif]
